# Cuboids Prevail When Unraveling the Influence of Microchip Geometry on Macrophage Interactions and Metabolic Responses

**DOI:** 10.1021/acsbiomaterials.4c00849

**Published:** 2024-08-21

**Authors:** Gordon Bruce, Saman Bagherpour, Marta Duch, José Antonio Plaza, Snow Stolnik, Lluïsa Pérez-García

**Affiliations:** † Division of Advanced Materials and Healthcare Technologies, School of Pharmacy, University of Nottingham, Nottingham NG7 2RD, U.K.; ‡ Departament de Farmacologia, Toxicologia i Química Terapèutica, Facultat de Farmàcia i Ciències de l’Alimentació, Universitat de Barcelona (UB), Av. Joan XXIII 27-31, 08028 Barcelona, Spain; § Institut de Nanociència i Nanotecnologia (IN2UB), Universitat de Barcelona (UB), 08028 Barcelona, Spain; ∥ Instituto de Microelectrónica de Barcelona IMB-CNM (CSIC), Campus UAB, Cerdanyola del Vallès, Barcelona 08193, Spain; ⊥ Division of Regenerative Medicine and Cellular Therapies, School of Pharmacy, University of Nottingham, Nottingham NG7 2RD, U.K.

**Keywords:** cuboid-shaped polysilicon microchips, side-scattering imaging flow cytometry, macrophages, surface-bound microchips, cell internalization, metabolic response, cytotoxicity

## Abstract

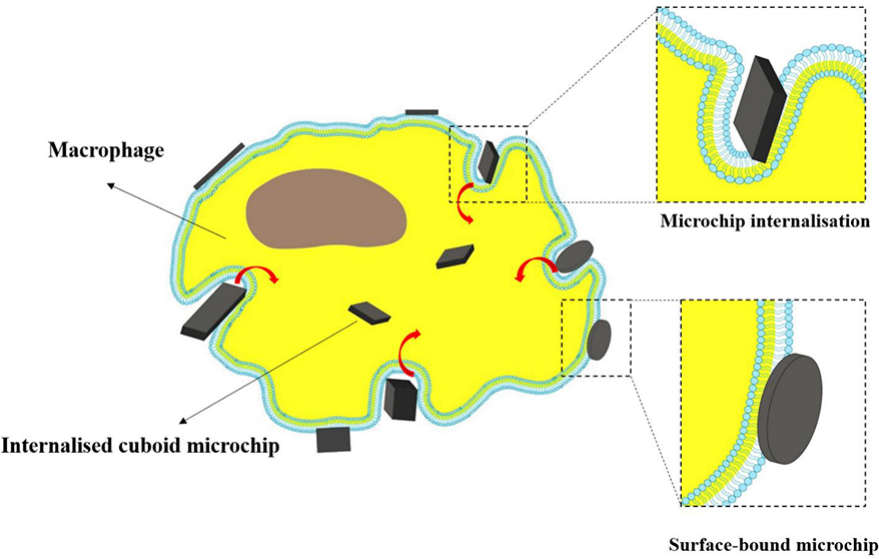

Drug delivery advances rely on using nano- and microsized carriers to transfer therapeutic molecules, although challenges persist in increasing the availability of new and even approved pharmaceutical products. Particle shape, a critical determinant in how these carriers distribute within the body after administration, raises opportunities of using, for instance, micrometer-sized nonspherical particles for vascular targeting and thereby creating new prospects for precise drug delivery to specific targeted areas. The versatility of polycrystalline silicon microfabrication allows for significant variation in the size and shape of microchips, and so, in the current work, photolithography was employed to create differently shaped polysilicon microchips, including cuboids, cubes, bars, and cylinders, to explore the influence of particle shape on cellular interactions. These microchips with different shapes and lateral dimensions, accounting for surface areas in the range of ca. 15 to 120 μm^2^ and corresponding total volumes of 0.4 to 27 μm^3^, serve as ideal models for investigating their interactions with macrophages with diameters of ca. 20 μm. Side-scattering imaging flow cytometry was employed for studying the interaction of label-free prepared microchips with RAW 264.7 macrophages. Using a dose of 3 microchips per cell, results show that cuboids exhibit the highest cellular association (ca. 25%) and uptake (ca. 20%), suggesting their potential as efficient carriers for targeted drug delivery to macrophages. Conversely, similarly sized cylinders and bar-shaped microchips exhibit lower uptakes of about 8% and about 6%, respectively, indicating potential benefits in evading macrophage recognition. On average, 1–1.5 microchips were internalized, and ca. 1 microchip was surface-bound per cell, with cuboids showing the higher values overall. Macrophages respond to microchips by increasing their metabolic activity and releasing low levels of intracellular enzymes, indicating reduced toxicity. Interestingly, increasing the particle dose enhances macrophage metabolic activity without significantly affecting enzyme release.

## 1 Introduction

Drug delivery can be significantly enhanced by using nano- and microsized carriers that transport therapeutic substances. The research in this field has significantly increased in recent years, but there are still several challenges to overcome in order to expand the availability of pharmaceutical products^1^ and the design of novel drug delivery systems. Various characteristics of particles, such as their size, shape, surface chemistry, charge, and mechanical properties, have been found to impact how they are taken up by cells. Some studies have compared the effect of particle size-mainly for spherical particles-on internalization into macrophages^2,3^ with significant distinction between the number of particles and the total surface area of particles ingested per cell; one key example showed that even when there was a higher number of 430 nm diameter particles internalized than 1.9 μm diameter particles, the total surface area of particles inside the cells was higher for the larger particles, which equates to a higher intracellular dose in the context of drug delivery.^4^

The advent of novel methods for fabricating particles of various morphologies has also resulted in increased interest in assessing the impact of particle shape on cellular interactions.^5^ The majority of studies assessing the effect of particle shape has relied on particles with nanosized dimensions owing to their potential use in systemic drug delivery as opposed to micron-sized particles which tend to be cleared faster from the body.^6^ Herd et al. examined the association of silica “worms” (232 × 1348 nm^2^) and spheres (diameter 178 nm) by RAW 264.7 using flow cytometry, showing that spheres exhibited a higher cellular association than worms.^7^ In contrast, Huang et al. found that rod shaped mesoporous silica particles (either 110 × 240 nm^2^ or 110 × 450 nm^2^) had a higher association with A375 epithelial cells than spherical particles (d = 100 nm).^8^ Regarding the interaction of microparticles with cells, Lu et al. studied the internalization of CdTe-quantum dot microcomposites of spherical (diameter 1.85 μm), rod (2.5 × 1.2 × 1.0 μm^3^), and needle (8.5 × 0.3 μm^2^) morphologies by RAW 264.7 cells.^9^ Spheres were internalized by a higher proportion of cells than both rod and needle-shaped particles with needle-shaped particles hardly being internalized by any cells. Kozlovskaya et al. also compared uptake of hydrogel capsules of spherical (diameter 1.8 μm) and discoidal (3.6 × 1.2 × 0.59 μm^3^) microparticles by J774A.1, HMVEC, and 4T1 cells,^10^ showing that more spheres were taken up per cell than discoidal particles in all cell lines.

The shape of particles has also been identified as a crucial factor influencing how particles distribute within the body following administration. Researchers have particularly focused on the behavior of micron-sized particles with various shapes in fluid flow.^11,12^ Due to the hydrodynamics of nonspherical microparticles in the bloodstream, these particles tend to migrate toward the walls of blood vessels. This observation has prompted scientists to investigate their potential for vascular targeting.^13,14^ Decuzzi et al. conducted experiments demonstrating variations in biodistribution among different particles, including uncoated spherical silica beads with diameters ranging from 700 nm to 3 μm, as well as uncoated quasi-hemispherical, discoidal, and cylindrical silicon-based particles.^15^ These findings present a new avenue to explore when considering the use of different particle shapes for targeting specific areas of the body.

In general, reports on the effects of nano- and microparticles’ shapes on cellular uptake are very mixed, and the interpretation of published studies is further complicated by the effects of other particle properties and different cell types. For example, the synthesis of nanoparticles of different shapes often involves the use of surfactants that, if not removed before uptake experiments, will have a large impact on the results. Despite being challenging, extending the current knowledge of how particle shape influences cellular uptake will lead to the design of more effective medicines. More specifically, since the majority of studies published thus far compare spherical and rod-shaped particles, the study of a more diverse array of particle shapes is warranted.

Polysilicon microchips of different morphologies manufactured by photolithography have been used by our group for a number of purposes. For instance, polysilicon barcodes have been used for cellular tracking in assisted reproductive process.^16^ More recently, polysilicon microdevices were fabricated to measure changes in intracellular pressure and to measure intracellular mechanical forces.^17^ Also, polysilicon star-shaped microchips were used to investigate how the presence of these internalized physical structures affects the cell cycle and leads to cell death.^18^ The internalization of polysilicon disk-shape microchips in THP-1 cells has been assessed by Fernandez-Rosas et al.^19^ However, a quantitative examination of particle uptake was not performed. Similar to polysilicon microparticles, monocrystalline silicon particles fabricated by a similar lithographic process have been assessed for their potential use as a drug delivery system in a number of studies by Ferrari’s group.^20^ Discoidal silicon microparticles have been also used as carriers for drug delivery purposes^21^ and chemotherapeutics.^22^

The above-mentioned examples exposed the difficulties of working with surface functionalized polysilicon microchips, resulting in qualitative results for investigating the interaction of this type of particles with living cells. On the other hand, labeling of polysilicon particles faces some difficulties resulting in a low level of functionalization homogeneity. Therefore, efficient quenching/labeling methods are difficult to employ for quantification of polysilicon particles cell internalization. Instead, side-scattering imaging flow cytometry (SSC-IFC) is a recently developed technique designed to address certain constraints by merging the spatial precision of microscopy with the rapid, high-throughput capabilities of flow cytometry, without the need of using fluorescent tags that could also alter the properties of the microchip’s surface.^23^

Some research studies have utilized particle side scattering to identify high refractive index inorganic particles within cells.^24^ This technique has been employed to observe cell interactions with nanoparticles of TiO_2_, Ag, Fe_3_O_4_, and Au, as well as carbon nanotubes.^25−27^ However, these studies have considered only nanometric-sized particles and have not examined the impact of various particle shapes on cell association quantitatively. Furthermore, side scattering has been applied to detect porous silicon microparticles in human umbilical vein endothelial cells, but they have only studied hemispherical and discoidal shapes.^28^

To the best of our knowledge, the effect of polysilicon microchips’ shape on internalization by macrophages has not been studied. In this work, label free polysilicon microchips serve as a model particle to investigate the effect of microchips’ shape on interactions with macrophages. Polysilicon microchips were fabricated in cuboid, cube, bar, and cylinder shapes and designed with lateral dimensions of 3 to 15 μm length, 3 μm width, 0.05 to 3 μm thickness, or 3 to 4 μm diameter in the case of cylinders so that all had at least one lateral dimension of ca. 3 μm as it has been shown that RAW 264.7 cells (ca. 20 μm in diameter) were able to internalize microchips within these dimensions. The chosen particle shapes allow for the effect of factors such as particle length, thickness, and curvature on the macrophage interaction to be examined. The interaction of label free polysilicon microchips with macrophages is investigated by SSC-IFC followed by the examination of cellular metabolism and toxicity in response to the various particle shapes.

## 2 Materials and Methods

### 2.1 Materials

Rhodamine B isothiocyanate (RBITC, 283924), 4-(2-hydroxyethyl)-1-piperazineethanesulfonic acid (HEPES H0887), Hank’s Balanced Salt Solution (HBSS, H8264), Dulbecco’s Modified Eagle Medium (DMEM, D546), Foetal bovine serum (FBS, F7524), l-glutamine (G7513), Triton X-100, Penicillin/Streptomycin, LDH assay kit, and 4-methyl umbelliferyl-β-d-glucuronide hydrate (MUG) were purchased from Sigma-Aldrich. The AQueous One solution cell proliferation assay (MTS reagent) was purchased from Promega UK. 11-Aminoundecyltriethoxysilane (AUTES, S25045) was purchased from Fluorochem (UK). H_2_SO_4_ (98%), NH_4_OH (20%), acetone, and ethanol were purchased from Fisher Scientific. Formaldehyde 4% and dimethyl sulfoxide (DMSO) were purchased from VWR. Phosphate-buffered saline (PBS) tablets were obtained from Oxoid Ltd. (UK). Tissue culture treated 75 cm^2^ (T-75) cell culture flasks were purchased from Corning Life Sciences (Holland). Accutase was purchased from Fisher Scientific. Milli-Q water was produced by a Milli-Q plus system from Millipore.

### 2.2 General Methods

Flow cytometry was performed using an Amnis imagestream^X^ MKII imaging flow cytometer in a standard configuration with 40× magnification. Illumination settings: Brightfield LED 30.75 mW, 642 nm laser 150 mW, 785 nm laser 1.25 mW. Data was acquired using INSPIRE software with a minimum of 500 cells per sample (typically > 1000 per sample). Data was analyzed using IDEAS software. Brightfield and fluorescence images were acquired using a Nikon Eclipse TiU fluorescence microscope. For fluorescence imaging of RBITC labeled samples, exposure time was kept constant (1 s) λ_ex_ = 550 nm and λ_em_ > 590 nm. Images were processed using ImageJ^29^ to produce fluorescence surface plots and measure median fluorescence intensity values. Six microchips were analyzed for each sample.

### 2.3 Polysilicon Microchip Fabrication

**Scheme 1:**
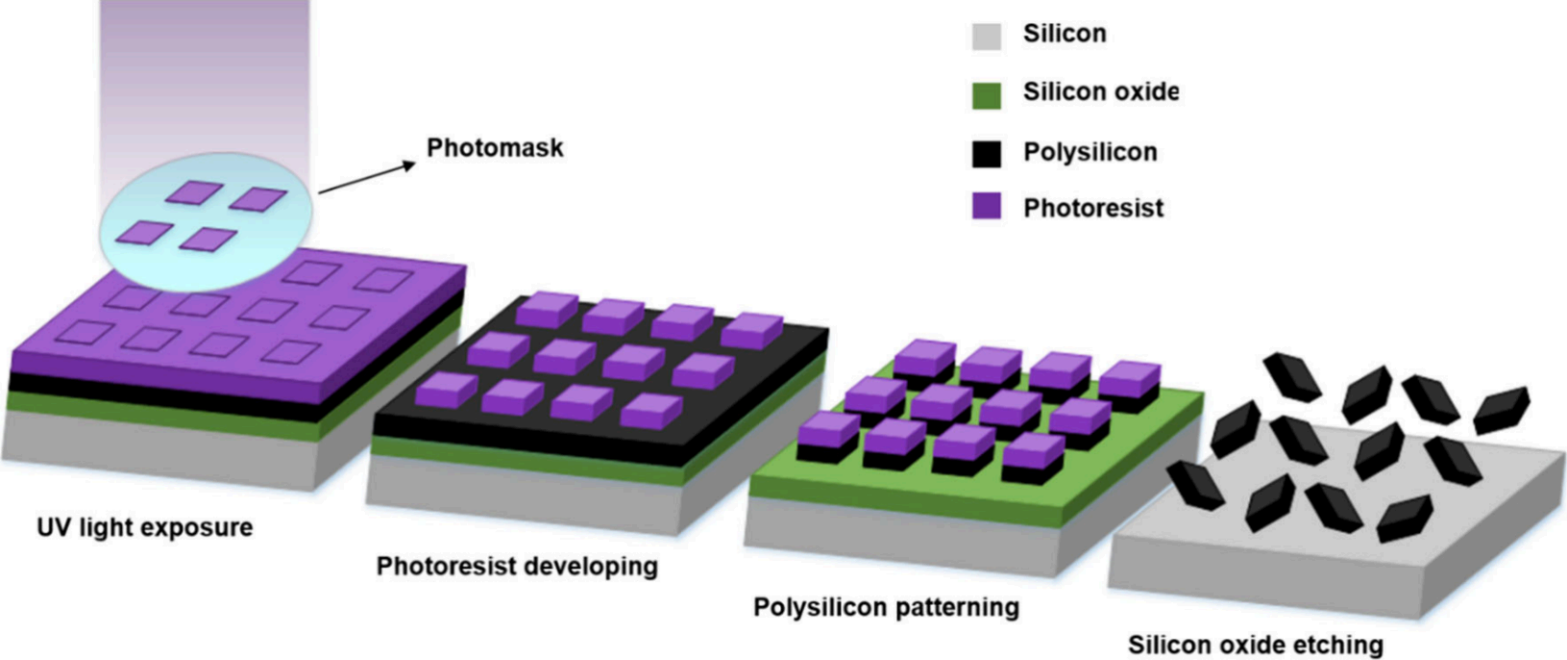
Schematic Fabrication Process of Polysilicon Microchips by Photolithography

Microchips were fabricated using silicon-based technologies based on a photolithography technique (Scheme 1). The silicon oxide layer was grown as a sacrificial layer with a thickness of 1 μm on the surface of a silicon wafer used as a substrate. Subsequently, a polysilicon layer was deposited as a structural layer with a thickness of either 0.05 or 0.5 μm using low-pressure chemical vapor deposition (LPCVD) on top of the silicon oxide layer. Next, a photoresist layer was spun onto the polysilicon layer and exposed to UV light for defining the microchips. The polysilicon layer was then subjected to a dry etching process to pattern the chips, and subsequently, the photoresist layer was removed. Finally, the sacrificial etching of the silicon oxide layer was performed to release the microchips. It is worth mentioning that Scheme 1 provides a general example of a microfabrication process for one type of microchip. The rest of the microchips are made similarly by changing the thickness of the polysilicon layer. SEM was performed on a LEO 1530 ZEISS instrument, and the images were analyzed to measure particle dimensions using ImageJ. Microchips were stored and transported in microcentrifuge tubes in ethanol at room temperature.

### 2.4 Label Free Detection of Polysilicon Microchips by Light Scattering

Microchips were assessed for their ability to scatter the 642 nm laser in an Amnis imagestream^X^ MKII imaging flow cytometer. The use of 642 nm light for detection is indeed inherent to the imaging flow cytometer employed for the light scattering, as it is the standard wavelength utilized by the device to detect scattering. Different polysilicon microchips with the same initial concentrations were suspended in 1% HEPES in HBSS, and scattering intensity histograms of each particle type were compared with the scattering of RAW 264.7 cells to see which microchips had sufficiently high scattering to be able to discriminate cells without microchips from cells with microchips.

### 2.5 Quantification of Polysilicon Particle Uptake by RAW 264.7 Cells by Flow Cytometry

#### Preparation of Microchips and Cells for Flow Cytometry

Microchips stored in ethanol in microcentrifuge tubes were centrifuged at 5700 rpm (6175 G) for 10 min. From this point on, all work was carried out inside a sterile cell culture hood to maintain sterility. The EtOH supernatant was removed, and the microchips were suspended in 1 mL of buffer comprising 1% HEPES in HBSS. Microchips were counted using a hemocytometer (Scientific Laboratory Supplies (UK)), and the required volume of each particle stock containing 300,000 microchips was transferred to fresh microcentrifuge tubes. The volume of each microcentrifuge tube was then increased to 1 mL with the required volume of 1% HEPES in HBSS. Particle suspensions were warmed to 37 °C before addition to cells.

RAW 264.7 cells were harvested once they reached 60–80% confluency by scraping as described in the Supporting Information (Routine cell culture methods). 100,000 cells in culture media were seeded per well into a 12 well plate and incubated overnight at 37 °C, 5% CO_2_, 95% humidity. Culture media were removed by aspiration, and the cells were washed with 1 mL of prewarmed (37 °C) PBS. PBS was removed, and 1 mL of prewarmed particle suspensions was applied at a ratio of 3 microchips per cell. Cells were incubated for 4 h after which time the particle suspensions were removed, and the cells were washed with warm HBSS (3 × 1 mL). HBSS was removed from each well by aspiration, 250 μL of accutase was added to each well, and the cells were placed in an incubator for 5 min to detach cells. 250 μL of HBSS was added to each well, and the total volume of each well was transferred to fresh microcentrifuge tubes. Samples were centrifuged at 250 G for 5 min, and the supernatants were removed. The cells were suspended in 250 μL of 4% formaldehyde in PBS for 20 min before being centrifuged for 5 min (250 G), and the supernatant was removed. Fixed cells were suspended in 50 μL of HBSS and stored at 4 °C until analysis by flow cytometry. Samples were always analyzed within 1 week of sample preparation.

#### Quantification of Particle Internalization by Imaging Flow Cytometry (IFC)

Single cell populations were first determined by plotting a scatter plot of the area against the aspect ratio. Scattering intensity histograms of single cells were plotted to distinguish particle associated and nonassociated cells as shown in Figure S1. Cells with internal microchips were distinguished from cells with surface-bound microchips using imaging flow cytometry and light scattering to identify the location of the particle with respect to the cell (Figure S2). In order to distinguish surface bound microchips from internalized microchips, a cell mask was created. This mask was eroded to exclude the cell membrane using the adaptive erode feature with an adaptive erode coefficient of 80. This defines an area specific to each cell outside of which microchips are considered surface bound by considering the ratio of the scattered light inside the mask to the scattered light of the entire cell. This analysis gives each image an internalization score where a positive score indicates the particle is inside the cell and a negative score indicates the particle is surface bound. A histogram displaying the internalization scores is produced, and regions were defined as “External” and “Internal”.

### 2.6 Preparation of Microchips and Cells for MTS, LDH, and Glucuronidase Assays

Microchips stored in EtOH in microcentrifuge tubes were centrifuged at 5700 rpm (6175 G) for 10 min. From this point on, all work was carried out inside a sterile cell culture hood to maintain sterility. The EtOH supernatant was removed, and the microchips were suspended in 1 mL of buffer comprising 1% HEPES in HBSS. Microchips were counted using a hemocytometer, and the required volume of each particle stock was transferred to fresh microcentrifuge tubes. The volume of each microcentrifuge tube was made up of 500 μL with the required volume of 1% HEPES in HBSS. Particle suspensions were warmed to 37 °C before addition to cells.

RAW 264.7 cells were harvested once they reached 60–80% confluency by scraping as described above (Routine cell culture methods in the Supporting Information). 10,000 cells in 150 μL of culture media were seeded per well into a clear 96 well plate and incubated overnight at 37 °C, 5% CO_2_, 95% humidity. Culture media were removed by aspiration, and the cells were washed with 200 μL of prewarmed (37 °C) PBS. PBS was removed, and 150 μL of prewarmed particle suspensions was applied to each well. 150 μL of 1% HEPES in HBSS was applied as a negative control, and 0.1% Triton X-100 in PBS was applied as a positive control. Cells were incubated for 4 h after which 50 μL of the cell-conditioned buffer (the buffer, which has been exposed to the cells and therefore contains anything released by the cells, such as enzymes, etc.) was removed from each well and transferred to a fresh clear 96-well plate for the LDH assay. Additionally, 50 μL of the cell-conditioned buffer was transferred to a fresh black 96-well plate for the glucuronidase assay. The remaining cell-conditioned buffer was removed by aspiration, and the cells were washed with 3 × 150 μL of prewarmed PBS.

### 2.7 MTS, LDH, and Glucuronidase Assays

Microchips and cells were prepared according to the procedure explained in the Supporting Information. The MTS assay measures cell metabolic activity by using a tetrazolium dye and an electron coupling reagent, resulting in a colored formazan product whose quantity is proportional to the number of active cells, determined by measuring absorbance at 492 nm. After removal of the cell-conditioned buffer and washings with 3 × 150 μL of PBS, the remaining PBS was aspirated, and 20 μL of the MTS reagent in 100 μL of culture medium was added to each well. Cells were incubated at 37 °C, 95% humidity, 5% CO_2_ for 2 h, after which time the absorbance at 492 nm of each well was measured using a TECAN spark microplate reader. Relative metabolic activity was calculated with respect to the negative control by using eq 1
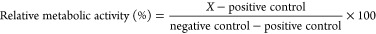
1where *X* is the absorbance of the sample well.

The LDH assay also measures cell viability by quantifying LDH release, an indicator of cell death resulting from membrane damage, through the reduction of a tetrazolium reagent to a colored formazan product with absorbance at 492 nm providing a measure of relative LDH release. To the clear 96 well plate containing 50 μL of the cell-conditioned buffer per well was added 100 μL of the LDH reagent, and the well plate was left for 2 h at room temperature while being protected from light. After this time, the absorbance at 492 nm was measured by using a TECAN spark microplate reader. Relative LDH release with respect to the positive control was calculated using eq 2
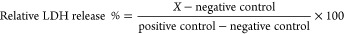
2where *X* is absorbance of the sample well.

The nonfluorescent substrate 4-methylumbelliferyl-β-d-glucuronide hydrate (MUG) is cleaved by β-glucuronidase, resulting in the production of fluorescent 4-methylumbelliferone with excitation at 372 nm and emission at 445 nm, allowing for measurement of lysosomal enzyme release into the culture media based on the proportional fluorescent signal. MUG was dissolved in sodium acetate buffer 0.1 M and pH 4.5 to a final concentration of 100 μM. To the black 96 well plate containing 50 μL of the cell-conditioned buffer per well was added 100 μL of the MUG solution per well. The plate was then protected from light and incubated for 2 h at 37 °C, 95% humidity, 5% CO_2_. After this time, 10 μL of NH_4_OH per well was added to terminate the reaction and increase the fluorescent signal. The fluorescence signal at 460 nm was then measured after excitation at 360 nm using a TECAN spark microplate reader, which were the closest options for excitation and emission according to the excitation and emission of the produced fluorescent 4-methylumbelliferone. Relative β-glucuronidase release with respect to the positive control was then calculated using eq 3
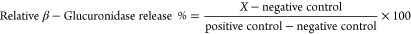
3where *X* is emission of the sample well.

## 3 Results and Discussion

### 3.1 Polysilicon Microchip Characterization

Microfabricated microchips are good candidates for microcarriers due to their controlled multifunctionalization, precise size and shape, and high surface area. These features enhance their versatility and functionality, allowing tailored designs for biological uses. They are easier to observe microscopically, exhibit prolonged retention in cells, and often accumulate in targeted tissues, making them reliable for biomedical applications.

**Figure 1:**
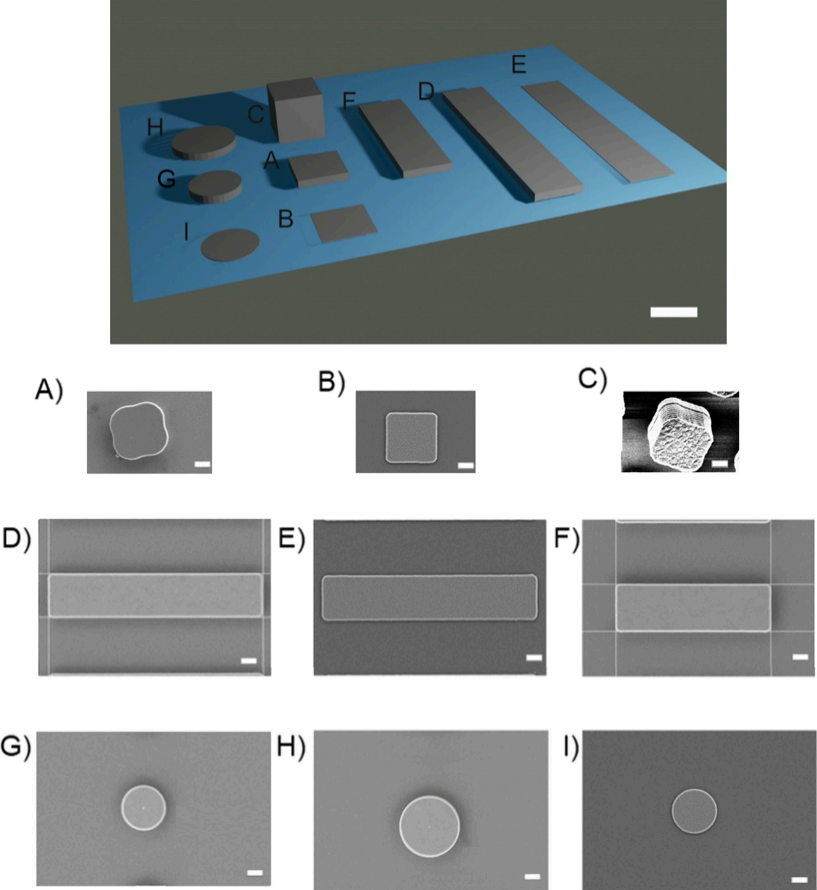
(Top) 3D Schematics of designed microchips (scale bar 3 μm). (Bottom) SEM images of the polysilicon microchips. A) 3 × 3 × 0.5 μm^3^ cuboids, B) 3 × 3 × 0.05 μm^3^ cuboids, C) 3 × 3 × 3 μm^3^ cubes, D) 3 × 15 × 0.5 μm^3^ bars, E) 3 × 15 × 0.05 μm^3^ bars, F) 3 × 10 × 0.5 μm^3^ bars, G) cylinders’ diameter 3 μm, thickness 0.5 μm, H) cylinders’ diameter 4 μm, thickness 0.5 μm, I) cylinders’ diameter 3 μm, thickness 0.05 μm (Scale bars = 1 μm).

Polysilicon microchips designed in different shapes and dimensions were fabricated using photolithography processes (Scheme 1) and were characterized by SEM imaging (Figure 1). The designs include different shapes including cuboids, bars, and cylinders, with different thicknesses and lateral dimensions. Figure 1 exhibits the SEM images of various types of fabricated microchips. Table 1 also shows the dimensions of the fabricated polysilicon microchips. As it has been demonstrated that the thickness of the microchips affects their internalization rate by cells, cuboid devices were first fabricated, which differ in their thickness ranging from 50 nm to 3 μm. Figures 1A and 1B show cuboids polysilicon microchips with the same length and width (3 μm) but different thicknesses. Figure 1C also exhibits the cube-type microchips with the same length, width, and thickness. Then, devices with the same width as the cuboids but with larger length as torpedo-like microchips were fabricated. Figure 1D-F shows the bar type of polysilicon microchips, which were fabricated with the same width (3 μm) and different height and length dimensions. Finally, in order to study if the corners of the chips can affect cell interaction, cylinder-shaped microchips (with the diameter of 3 to 4 μm) were fabricated as shown in Figure 1G-I. To enable RAW 264.7 cells to internalize microchips with these dimensions, shapes were always fabricated with at least one dimension of 3 μm (except for one of the cylinders, which is 4 μm).

**Table 1:**
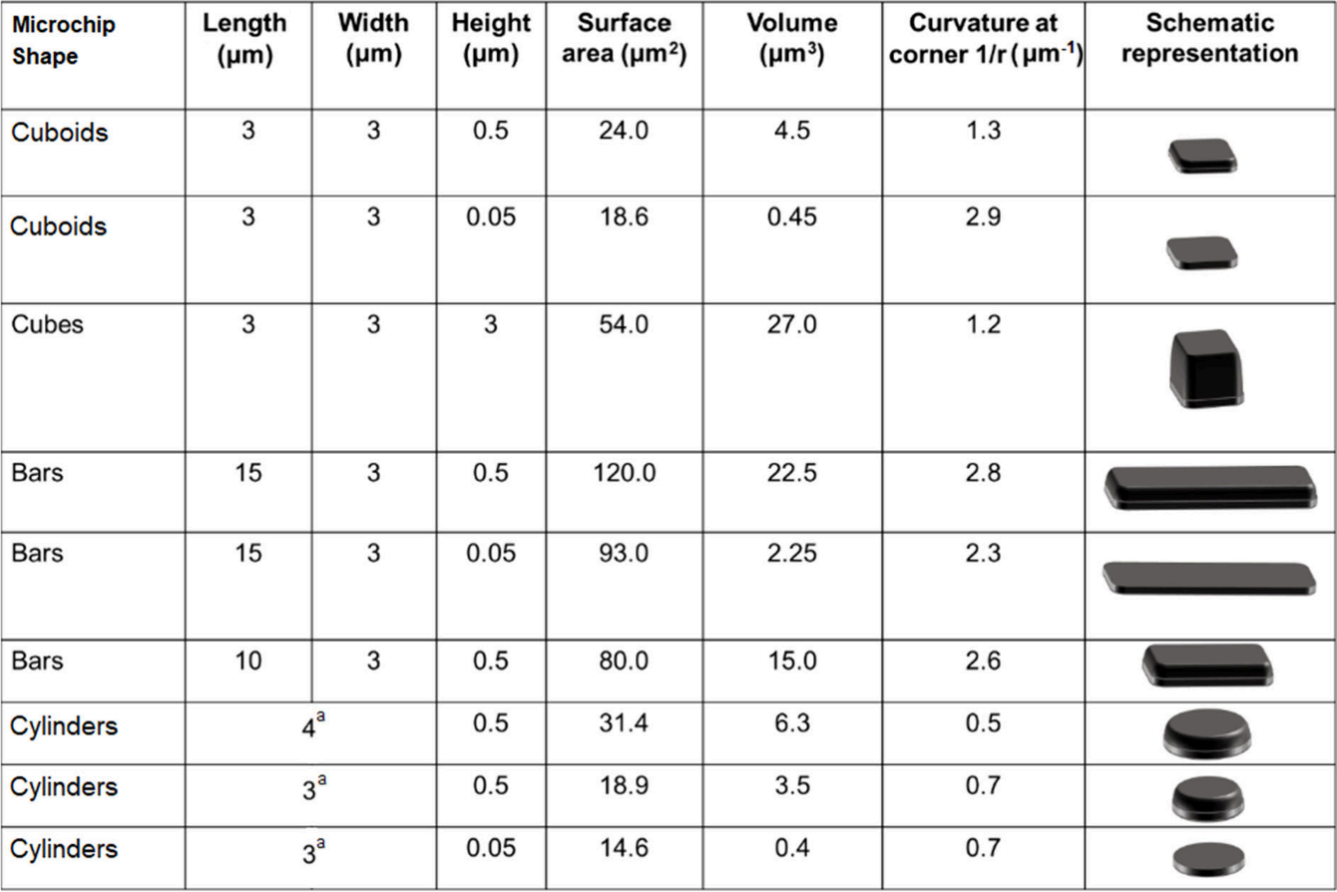
Microchips’ Shapes and Their Dimensions^a^

a For cylinders, these lateral dimensions correspond to their diameter.

### 3.2 Assessment of Particle Side Scattering

**Figure 2:**
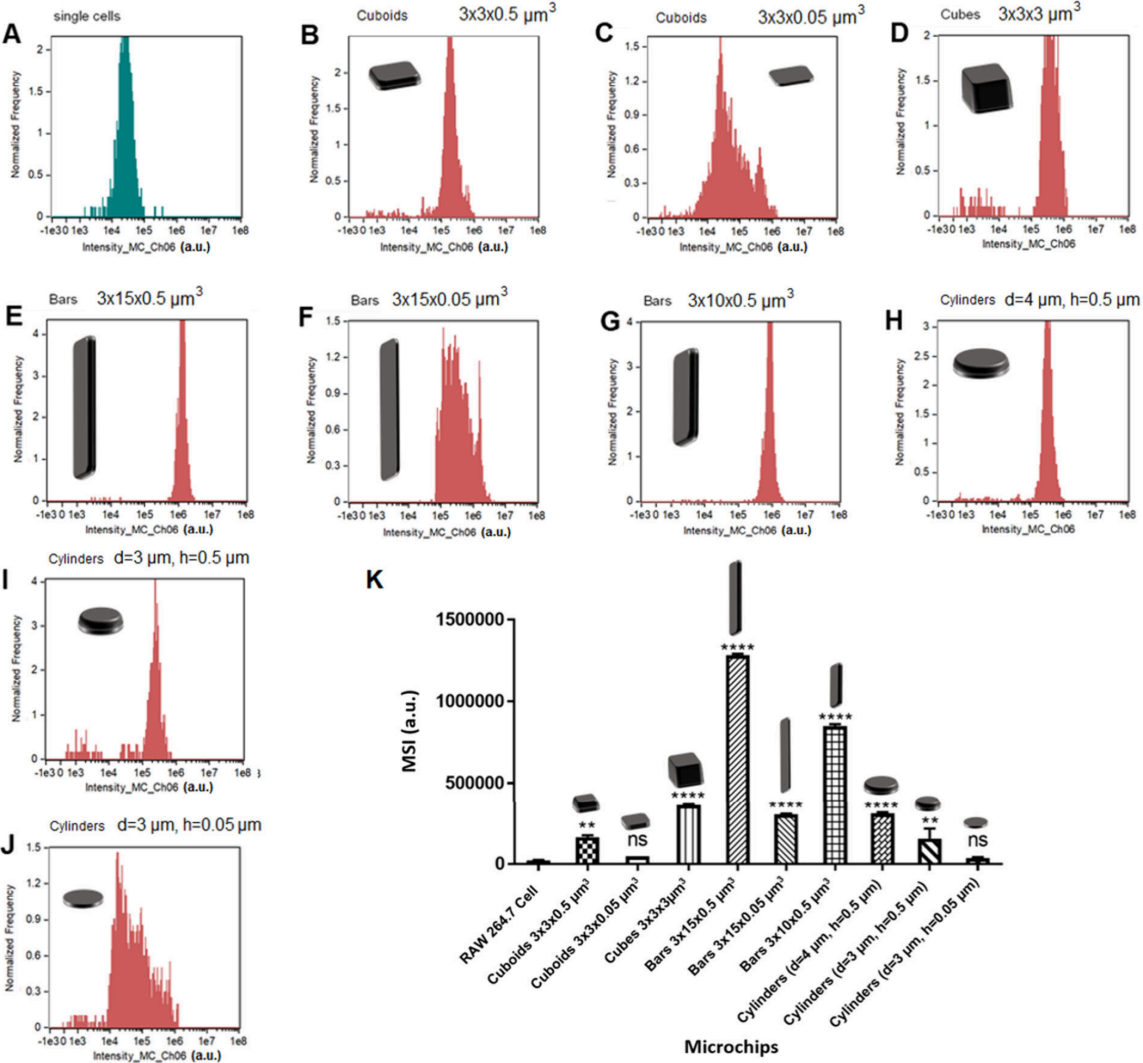
Scattering intensity histograms of RAW 264.7 cells and each particle shape at the same initial concentrations. A) RAW 264.7 cells, B) cuboids 3 × 3 × 0.5 μm^3^, C) cuboids 3 × 3 × 0.05 μm^3^, D) cubes 3 × 3 × 3 μm^3^, E) Bars 3 × 15 × 0.5 μm^3^, F) Bars 3 × 15 × 0.05 μm^3^, G) Bars 3 × 10 × 0.5 μm^3^, H) cylinders (diameter (d) = 4 μm, height (h) = 0.5 μm), I) cylinders (d = 3 μm, h = 0.5 μm), J) cylinders (d = 3 μm, h = 0.05 μm). K) Summary of median scattering intensity (MSI) values for RAW 264.7 cells and each particle shape. A) Particle MSI values compared with RAW 264.7 cells. * indicates a statistically significant difference between particle scattering and RAW 264.7 cell scattering as analyzed by one-way ANOVA with Dunnet’s multiple comparisons (**p < 0.01, ****p < 0.0001, ns = not significant).

A label-free method of detecting polysilicon microchips was pursued which eliminates the difficulties related to labeling polysilicon microchips. In addition, it ensures that all microchips have the same surface chemistry to ensure their effect on cellular interaction between the different particle shapes is negligible. Side scatter is heavily dependent on both refractive index and particle shape, and so each particle shape was assessed and compared with cellular side scatter to ensure that the two could be distinguished in a mixed population. The scattering intensity histograms of each particle shape are shown in Figure 2. RAW 264.7 cells displayed a median scattering intensity (MSI) of 2.2 × 10^4^ ± 3.5 × 10^2^ a.u. (arbitrary unit) (Figure 2A), and so the MSI of microchips needed to be significantly higher than these values in order to unambiguously distinguish between particle associated and nonassociated cells. Figure 2K displays the MSI values of each particle shape. The data show that for the majority of particle shapes the scattering intensity was significantly higher than that of RAW 264.7 cells, meaning that they could be used in a label-free manner for uptake studies. However, this was not the case for 3 × 3 × 0.05 μm^3^ cuboids and 3 × 0.05 μm^3^ cylinders which both had similar MSI to that of RAW 264.7 cells. This is likely because of the thickness of the microchips (0.05 μm) which makes the microchips semitransparent and thus reduces the amount of light scattered. These microchips were therefore not suitable for use in cell uptake studies using this method. However, the cylinders with dimensions of 4 × 0.5 μm^3^ and 3 × 0.5 μm^3^ show considerable scattering intensity compared to macrophages, indicating their potential for utilization in cell uptake investigation. On the other hand, bars with dimensions of 3 × 15 × 0.5 μm^3^ exhibited maximum scattering compared to other types of fabricated microchips. Despite having the same thickness, bars with dimensions 3 × 15 × 0.05 μm^3^ were able to be distinguished from cells because of their length, although scattering was significantly reduced compared to 3 × 15 × 0.5 μm^3^ bars.

The scattering intensity showed a correlation with particle surface area (Figure S3 in the Supporting Information). In terms of measuring cellular uptake, this method of detection is advantageous because it avoids a chance of differences in surface labeling affecting the results. Studies have shown that having a higher amount of surface labeling can increase uptake,^30^ potentially due to the hydrophobicity of the dye molecules, and so by maintaining the uniformity of the particle surface chemistry across all particle shapes, the chance of erroneous results relating to this is removed.

### 3.3 Polysilicon Microchip Association with RAW 264.7 Macrophages

**Figure 3:**
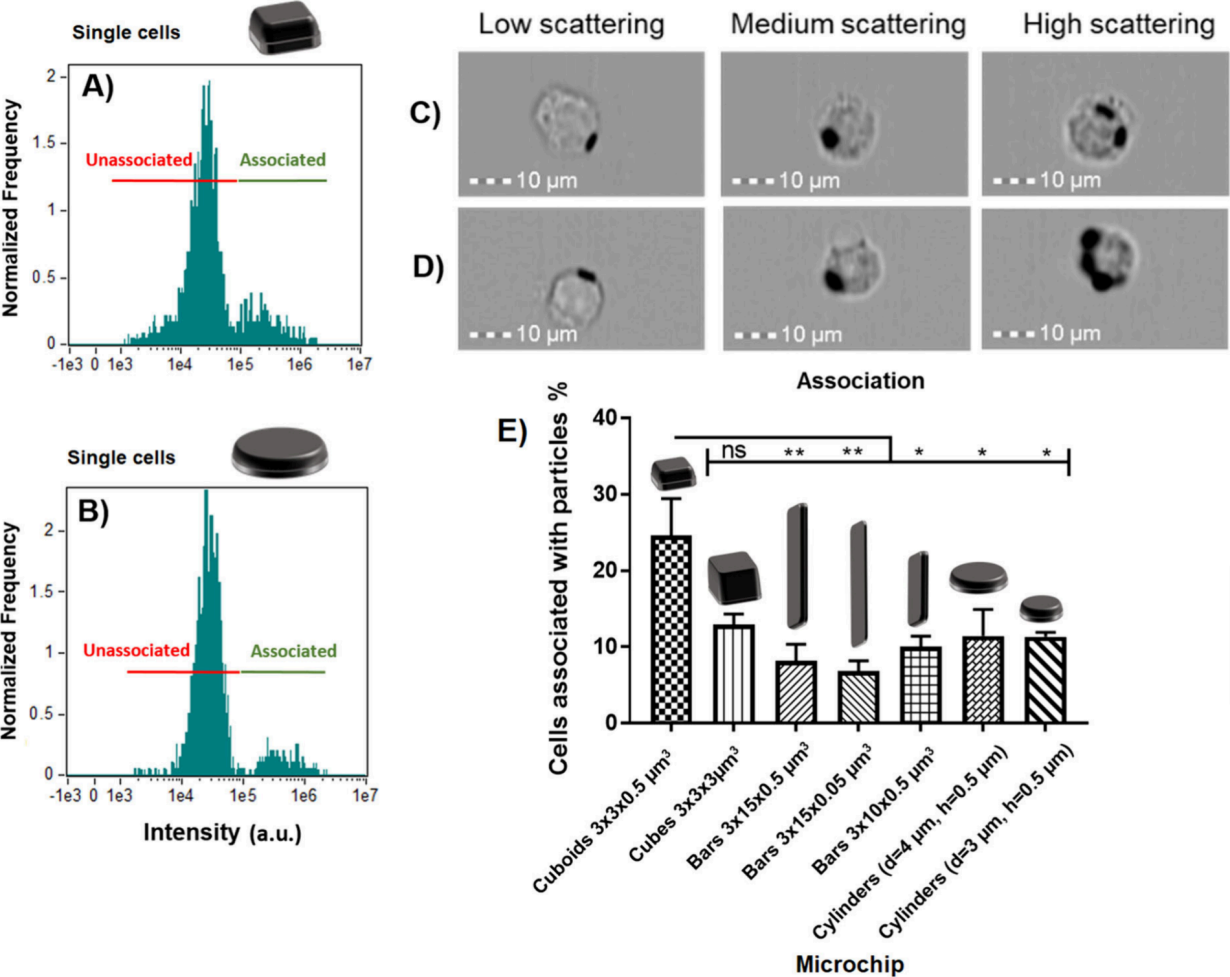
Side scattering intensity histograms of RAW 264.7 cells incubated with polysilicon microchips. A) Cuboids (3 × 3 × 0.5 μm^3^) and B) Cylinders (d = 4 μm h = 0.5 μm). Cell images from imaging flow cytometry across the range of scattering intensities for cells C) associated with cuboids (3 × 3 × 0.5 μm^3^) and D) cylinders (d = 4 μm h = 0.5 μm) polysilicon microchips. E) The percentage of cellular association of polysilicon microchips with RAW 264.7 cells. Statistical differences analyzed by one-way ANOVA with Tukey’s multiple comparisons test (* *p* < 0.05, ** *p* < 0.01, *** *p* < 0.001, **** *p* < 0.0001). *n* = 1, *N* = 3 ± SEM.

Side scattering intensity of RAW 264.7 cells that had been exposed to polysilicon microchips for 4 h was measured by imaging flow cytometry (SSC-IFC). Representative scattering intensity histograms for cuboids (3 × 3 × 0.5 μm^3^) and cylinders (d = 4 μm h = 0.5 μm) are shown in Figure 3A-B and for the rest of microchips in Figure S4. In each case, two clear populations of unassociated cells (lower scattering intensity) and cells associated with microchips (higher scattering intensity) can be clearly distinguished. This was confirmed by visual examination of the cell images in each population by looking at images across the intensity range of each population. After applying the gating for associated cells, images that had median scattering, as defined by the spectrum, were used for ’medium’. Images for ’low’ and ’high’ were taken from bins of particle-associated cells with the lowest and highest scattering intensity, respectively. Cell images from low (minimum), medium (median), and high (maximum) scattering intensity for each population for each sample are shown in Figure 3C–D and Figure S5 in the Supporting Information. As can be seen from the images of nonassociated cells, high scattering intensity relates to having a more granular appearance than those of low scattering. In particle-associated cells, higher scattering was indicative of more microchips associating with the cell.

The percentage of cells that are associated with microchips is shown in Figure 3E. 3 × 3 × 0.5 μm^3^ cuboids had the highest association (24.6 ± 4.8%) and was significantly higher than all other particle shapes apart from 3 × 3 × 3 μm^3^ cubes (12.9 ± 1.3%). There were no significant differences between 3 × 15 × 0.5 μm^3^ bars (8.1 ± 2.2%), 3 × 15 × 0.05 μm^3^ bars (6.8 ± 1.2%), 3 × 10 × 0.5 μm^3^ bars (10.0 ± 1.3%), cylinders with d = 4 μm and h = 0.5 μm (11.45 ± 3.2%), and cylinders with d = 4 μm and h = 0.5 μm (11.3 ± 0.7%). Despite having similar dimensions, there is a large difference between the association of 3 × 3 × 0.5 μm^3^ cuboids and 4 and 3 μm cylinders. This may point to a role for particle curvature in influencing the cellular association. Bar-shaped microchips showed the lowest association, and no effect of bar thickness (0.5 μm vs 0.05 μm) or length (15 μm vs 10 μm) was observed. Due to the large size of these microchips, it was not clear whether the macrophages would internalize the microchips or simply spread onto the particle surface in so-called frustrated phagocytosis.^31^

**Figure 4:**
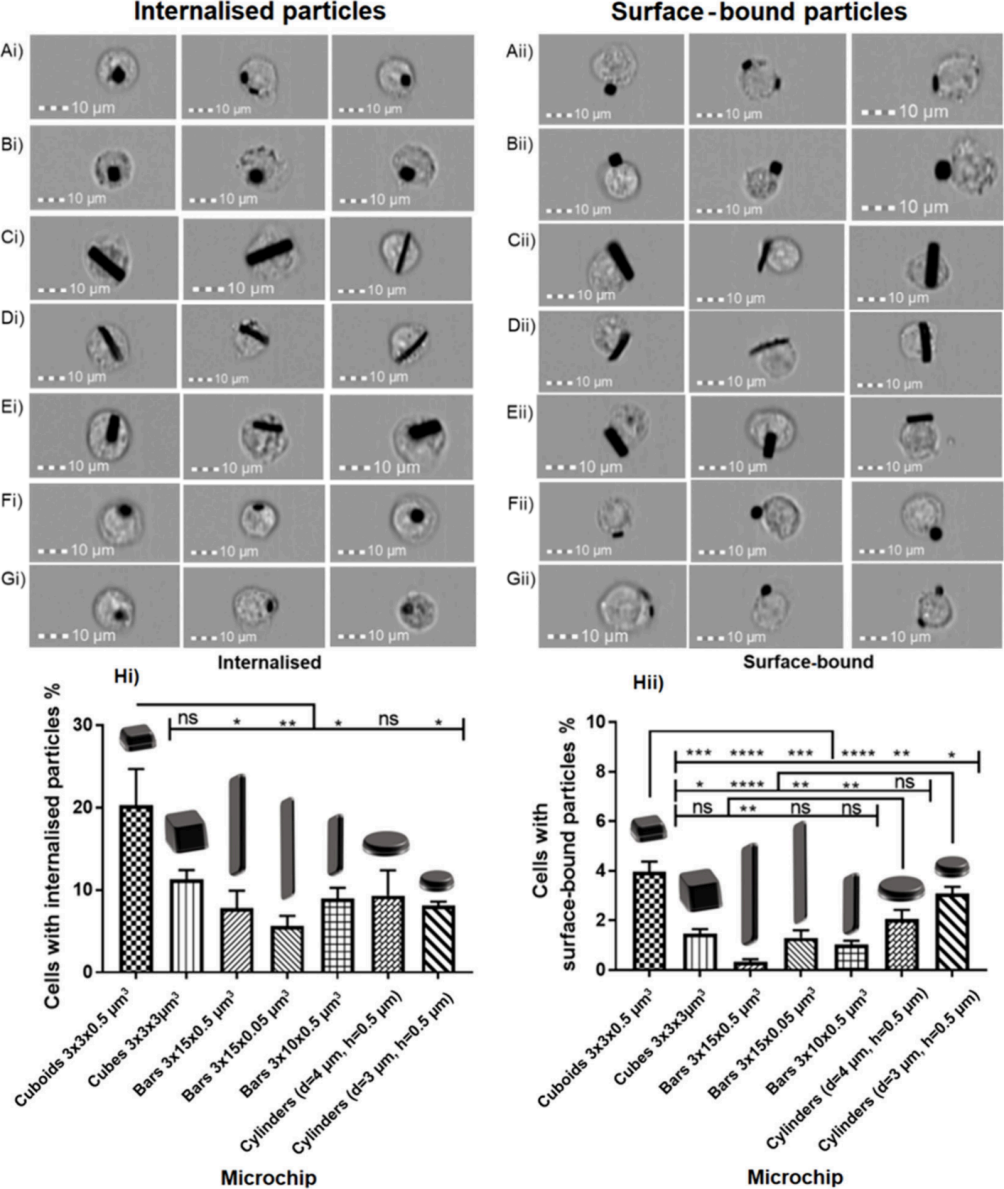
Cell images from the imaging flow cytometry of cells with internal and external microchips. A) Cuboids (3 × 3 × 0.5 μm^3^), B) Cubes (3 × 3 × 3 μm^3^), C) Bars (3 × 15 × 0.5 μm^3^), D) Bars (3 × 15 × 0.05 μm^3^), E) Bars (3 × 10 × 0.5 μm^3^), F) Cylinders (d = 4 μm h = 0.5 μm), G) Cylinders (d = 3 μm h = 0.5 μm). The percentage of cells with Hi) internalized microchips and Hii) surface-bound microchips. Statistical differences analyzed by one-way ANOVA with Tukey’s multiple comparisons test (* *p* < 0.05, ** *p* < 0.01, *** *p* < 0.001, **** *p* < 0.0001). *n* = 1, *N* = 3 ± SEM.

To examine this, imaging flow cytometry (IFC) was applied in order to assess the percentage of cells with internal microchips and the percentage of cells with surface-bound microchips. After application of the analysis, images of cells were assessed visually to check whether internal and surface-bound microchips with different orientations were distinguished (Figure 4A-G). For bar-shaped microchips, there were clear instances of particle internalization, whereby the shape of the cell is less circular and appears to have stretched to accommodate the presence of the internalized bar. Cells with surface-bound microchips could be distinguished from those with internalized microchips.

Figure 4Hi shows the percentage of cells that had internal microchips. Similar to the association data, 3 × 3 × 0.5 μm^3^ cuboids showed the highest internalization (20.3 ± 4.2%), although there were no significant differences for 3 × 3 × 3 μm^3^ cubes (11.2 ± 1.1%) or cylinders with d = 4 μm and h = 0.5 μm (9.3 ± 2.8%). Neither were significant differences in internalization between 3 × 15 × 0.5 μm^3^ bars (7.8 ± 1.9%), 3 × 15 × 0.05 μm^3^ bars (5.6 ± 1.1%), 3 × 10 × 0.5 μm^3^ bars (8.9 ± 1.2%), and cylinders with d = 3 μm and h = 0.5 μm (8.1 ± 0.3%). Figure 4Hii shows the percentage of cells that had surface-bound microchips. Here, more significant differences between the different particle shapes were observed. 3 × 3 × 0.5 μm^3^ cuboids showed significantly higher surface binding than all other shapes (3.9 ± 0.4%). Cylinders with d = 3 μm and h = 0.5 μm and cylinders with d = 4 μm and h = 0.5 μm showed statistically equivalent surface binding (3.1 ± 0.4% and 2.1 ± 0.3%, respectively). 3 × 3 × 3 μm^3^ cubes, 3 × 15 × 0.5 μm^3^ bars, 3 × 15 × 0.05 μm^3^ bars, and 3 × 10 × 0.5 μm^3^ bars showed the lowest surface binding (1.5 ± 0.4%, 0.3 ± 0.1%, 1.3 ± 0.3%, and 1.0 ± 0.2%, respectively). As the first stage of phagocytosis is binding of the cell membrane to the particle, the higher surface binding of cuboids tallies well with their increased uptake.

Low binding of cells to bar-shaped microchips could be the reason for lower internalization. The effect of microchip volume and surface area on cellular association, internalization, and surface binding was assessed to see if a correlation was present (Figure S6 in the Supporting Information). For cellular association (Figure S6A) and internalization (Figure S6B), there was a poor correlation with both particle surface area and particle volume. This indicates that differences in cellular association and internalization are not dependent on particle surface area or volume when different shapes of particle are administered to cells. For surface binding (Figure S6C), there was an inverse correlation with the particle surface area. A higher proportion of cells had surface bound microchips when the particle surface area was smaller. The number of microchips per cell was calculated by dividing the MSI of the cells with internal or surface-bound microchips by the MSI of the microchips; these data are shown in Figure S7. On average, between 1 and 1.5 microchips were internalized per cell, with individual values (in brackets) according to shape in the order: cylinders with d = 4 μm and h = 0.5 μm (1.42) ≥ cylinders with d = 3 μm and h = 0.5 μm (1.36) ∼ 3 × 3 × 0.5 μm^3^ cuboids (1.34) > 3 × 15 × 0.05 μm^3^ bars (1.16) ∼ 3 × 3 × 3 μm^3^ cubes (1.15) > 3 × 10 × 0.5 μm^3^ bars (0.82) > 3 × 15 × 0.5 μm^3^ bars (0.62) (Figure S7A). As for surface-bound microchips (Figure S7B), about 1 surface-bound particle per cell was estimated in the order: 3 × 15 × 0.05 μm^3^ bars (1.16) ∼ cylinders with d = 3 μm and h = 0.5 μm (1.15) ∼ 3 × 3 × 0.5 μm^3^ cuboids (1.11) = 3 × 3 × 3 μm^3^ cubes (1.15) ≥ cylinders with d = 4 μm and h = 0.5 μm (0.99) > 3 × 10 × 0.5 μm^3^ bars (0.47) > 3 × 15 × 0.5 μm^3^ bars (0.22). Overall, cuboids are the microchips at the front line for both cell surface binding and internalization, whereas thicker bars (0.5 μm) show lower performance. In comparison with the 3 × 15 × 0.05 μm^3^ bars, the calculated values for 3 × 15 × 0.5 μm^3^ bars and 3 × 10 × 0.5 μm^3^ bars are <1 because the scattering intensity values for the cells with microchips are lower than the MSI value of the microchips on their own. One possible explanation could be the orientation of the microchips. When the MSI of the microchips alone was measured for 3 × 15 × 0.5 μm^3^ bars and 3 × 10 × 0.5 μm^3^ bars, the microchips aligned with the flow, and so the MSI of the bars was calculated based on a single particle orientation. However, when the bars are present inside or on the surface of cells, the orientation is more random with respect to the detector, and so there is a disparity between the MSI of microchips alone and the microchips with cells. After manually reviewing the images of cells that were exposed to 3 × 15 × 0.5 μm^3^ bars, 91.4% (±3.9%) of cells that had internalized microchips had internalized 1 particle, and 100% of cells that had surface-bound microchips had 1 particle bound. For 3 × 10 × 0.5 μm bars, 75.9% (±8.1%) of cells with internal microchips had internalized 1 particle, and 93.7% (±5.4%) of cells with surface-bound microchips had 1 surface-bound particle. These results therefore suggest that, in line with the other particle shapes, the vast majority of cells was associated with 1 particle.

### 3.4 Effect of Particle Shape on Metabolic Activity, Toxicity, and Lysosomal Enzyme Release

#### MTS Assay

**Figure 5:**
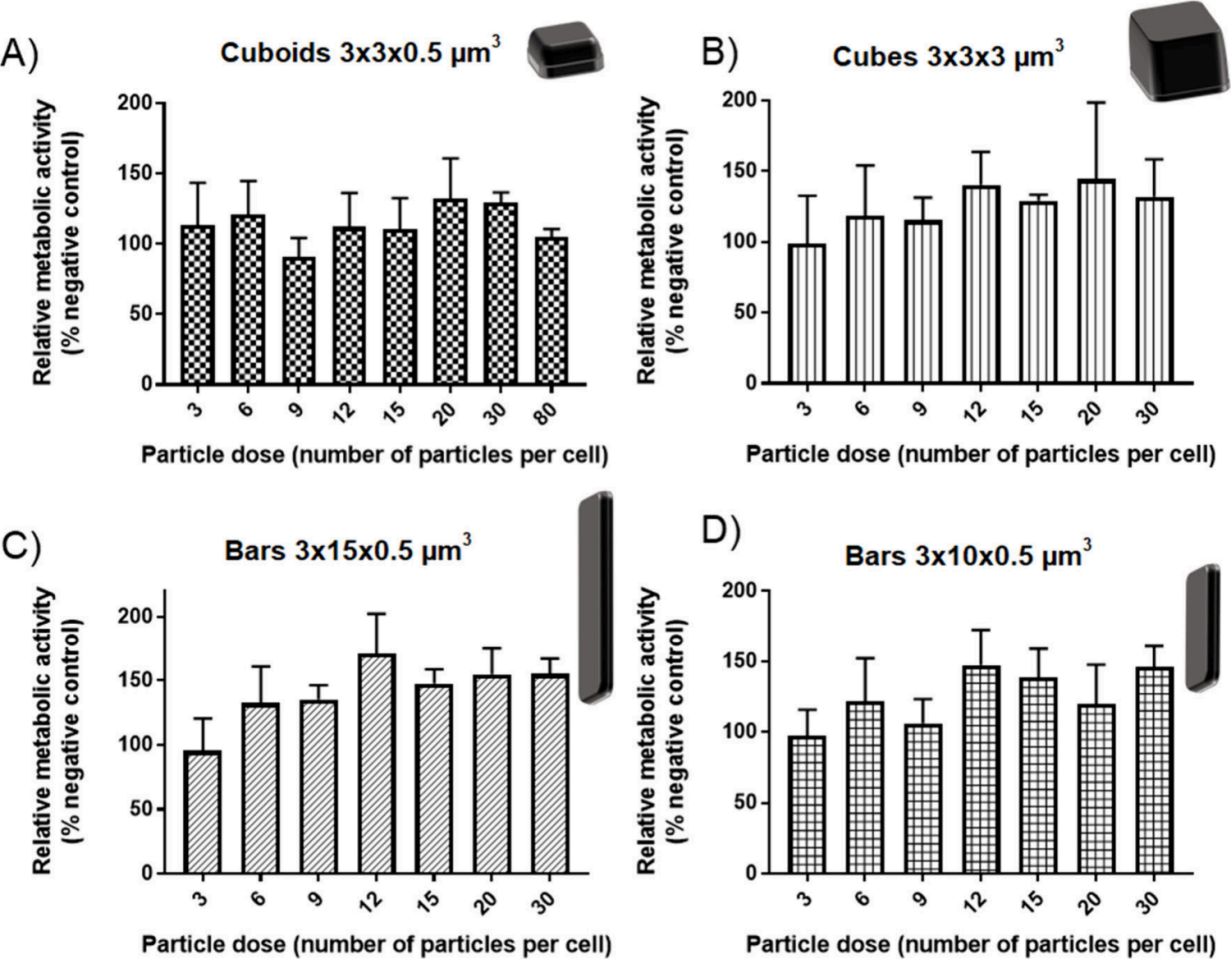
Effect of increasing the polysilicon particle dose on RAW 264.7 metabolic activity. A) Cuboids (3 × 3 × 0.5 μm^3^), B) Cubes (3 × 3 × 3 μm^3^), C) Bars (3 × 15 × 0.5 μm^3^), D) Bars (3 × 10 × 0.5 μm^3^). Relative metabolic activity was calculated with respect to cells treated with 1% HEPES in HBSS (positive control) and cells treated with 0.1% Triton X-100 (negative control). Data are expressed as a mean ± SD (*n* = 3).

The MTS assay was used to measure the metabolic activity of RAW 264.7 cells upon exposure to increasing ratios of polysilicon microchips. Upon reduction of the assay compound by metabolically active cells producing dehydrogenase, a colored formazan product is produced which can be detected by absorbance measurements, in a quantity that is proportional to the number of active cells, so used as a measure of toxicity. However, as phagocytosis is an energy-dependent process requiring actin polymerization to rearrange cellular structure, the ATP requirement is increased and changes in macrophage metabolism; for example, an increase in the rate of glycolysis occurs.^32^ Changes in cellular metabolic activity in response to phagocytosis of microchips were therefore assessed using the MTS assay. Polysilicon cuboids (3 × 3 × 0.5 μm^3^), cubes (3 × 3 × 3 μm^3^), bars (3 × 15 × 0.5 μm^3^), and bars (3 × 10 × 0.5 μm^3^) were applied to RAW 264.7 macrophages, and the effect on cellular metabolic activity was studied (Figure 5). The maximum dose for cubes and bars was 30 microchips per cell, while for cuboids it was 80 microchips per cell.

The metabolic activity followed a similar trend for each particle shape. Cellular metabolism increased compared to that of cells treated with 1% HEPES in HBSS. Maximum metabolic activity of cells treated with polysilicon microchips was around 140–150% of control cells and was achieved at different particle doses. For cuboids and cubes, this occurred at 20 microchips per cell (10 μg/mL and 21 μg/mL respectively), and for bar shaped microchips 3 × 15 × 0.5 μm^3^ and 3 × 10 × 0.5 μm^3^, this occurred at 12 microchips per cell (31 μg/mL and 21 μg/mL respectively).

The increase in metabolic activity could be indicative of the increased energy requirement of the cells internalizing particles. As seen in the uptake data, between 5% and 20% of cells are likely to take up particles, which might explain why the increase in activity is not more pronounced at lower doses. The increases in metabolic activity do not match the uptake data; for example, the largest increase was seen with bar shaped particles which also had the lowest uptake. This may indicate that these bar shaped microchips required a disproportionately large increase in metabolic activity upon internalization.

#### LDH Assay and Release of Lysosomal Enzyme Glucuronidase Evaluation

Lactate dehydrogenase (LDH) is an intracellular enzyme which is present in the cytoplasm of cells and is only released extracellularly when the cellular membrane is damaged, so the LDH assay can be used to measure the toxicity of nano- and microparticles.^33^ Polysilicon microchips were applied at increasing doses, and the amount of LDH in the supernatant was measured compared with cells that were treated with 1% HEPES in HBSS (negative control, 0%) and cells treated with 0.1% Triton X-100 (positive control, 100%). The data is shown in Figure S8.

Low levels (5–10%) of LDH were released for each particle type for doses between 3 and 80 microchips per cell for cuboids, 3–30 for cubes, 3–20 for 3 × 15 × 0.5 μm^3^ bars, and 3–30 for 3 × 10 × 0.5 μm^3^ bars. For 3 × 15 × 0.5 μm^3^ bars, this increased to ∼17 ± 2% when the dose was increased to 30 particles per cell (78 μg/mL) indicating that at this dose an increasing number of cells were damaged by the particles. As in the case of the MTS assay, the EC_50_ value was not obtained for polysilicon particles.

The release of glucuronidase is indicative either of the cells’ requirement of additional membrane to engulf larger particles or damage to the cell membrane. The two can be distinguished by comparing glucuronidase release with LDH release which is only indicative of cell membrane permeability.^34^ Glucuronidase release was detected using 4-methyl umbelliferyl-β-d-glucuronide hydrate (MUG), a nonfluorescent molecule that becomes fluorescent upon degradation by glucuronidase, and the data are shown in Figure S9. The data for release of lysosomal glucuronidase follows a similar trend to LDH release. Low levels (∼5% of total glucuronidase) were released from each particle shape across the administered dose range. It does not appear from this data that a significant different release of lysosomal glucuronidase occurs in response to the different particle shapes used. However, the congruency with the LDH data suggests that particles are causing some degree of cell membrane damage, which causes the release of both LDH and glucuronidase at low levels.

Using similar doses of polycrystalline particles to those used in our study, Van Landuyt et al.^35^ studied the release of LDH and glucuronidase by NR8383 macrophages *in vitro* after exposure to quartz (of respirable size, but shape not well characterized) and showed similar enzyme release. It is worth mentioning that *in vitro* studies utilizing microparticles of different shapes have tended to focus on inhaled fibers with macrophage responses studied to assess potential toxicity predominantly by LDH release.^36^ In congruence with this study, low levels of LDH release were seen upon exposure to microchips at comparable doses, and very few differences were seen in LDH release by the different length microchips. The 3 × 15 × 0.5 μm^3^ bars used in this study were similar in length and elicited lower LDH release at the same dose which is not suggestive of frustrated phagocytosis.

On the other hand, it is unlikely that the microchips used in this study would cause a puncturing of the cell membrane in a manner similar to that described previously, as the dimensions are greater than those used by Watanabe.^37^ In the current work, it was shown that ∼10–20% of cells internalized microchips of each shape over the course of the experiment causing no significant cytotoxicity in the time scale of experiments (4 h). This may indicate that the assays in this study depict the early stages of low toxicity due to the increased metabolic activity and the release of low levels of intracellular enzymes. Therefore, direct disruption of the cellular membrane by contact with particles is not thought to be the cause of the toxicity.

There are few studies focusing on the shapes of microparticles and their uptake by macrophages. One of them looks at the difference in uptake of PLGA microparticles with spherical (diameter 2 μm) compared with equivalent particles stretched to decrease the aspect ratio to 0.2,^38^ showing that spherical particles were internalized to a greater extent than the stretched particles. Additionally, it has been demonstrated that macrophages are less able to internalize the high aspect ratio of worm-shaped particles (length ∼22 μm) than spherical particles (3 μm diameter).^39^ The phenomena was explained by low attachment of macrophages to the major axis of the particles (the tips of the particles). Given the results of these studies, it would be anticipated that the bar shaped microchips (3 × 15 × 0.5 μm^3^, 3 × 15 × 0.05 μm^3^, and 3 × 10 × 0.5 μm^3^) would therefore show drastically reduced uptake relative to microchips with a lower aspect ratio, which did not hold true in our study. This represents a clear advantage of the use of more rigid silicon-based materials over polymeric materials.

A number of studies suggest that macrophages are less capable of internalizing soft particles than those that are more rigid.^40,41^ However, this is therefore a confounding factor in such particle uptake studies, as it is difficult to separate from shape alone. No deformation of the majority of polysilicon microchips used in this study was observed, and so the effect of mechanical stiffness may be low. The exception to this is the 3 × 15 × 0.05 μm^3^ bars which were observed to bend slightly, most likely due to their thickness.

Cuboids (3 × 3 × 0.5 μm^3^) were associated with and internalized by significantly more macrophages than cylinders (diameter 3 μm). Given the similarity between these two particle sizes, similar binding and uptake characteristics would be expected. As far as we know, no published studies have compared the phagocytosis of these two shapes, although disk shaped particles have been used to be carried by macrophages to the site of action.^42^ There is therefore a precedent for the reduced uptake of disk-shaped particles, but no studies compare the uptake of cuboids and disks. Because cuboids have corners, there are points of high curvature compared to cylinders that may influence macrophage binding and internalization. Receptor clustering drives particle internalization during phagocytosis,^43^ suggesting that if the initial point of contact between particle and cell is at a point of high curvature receptor clustering could be more efficient and so drive more efficient phagocytosis. Further investigation of this is warranted by monitoring individual particle cell interactions.

## 4 Conclusions

The different shapes of polysilicon microchips had only small effects on their interactions with macrophages. In terms of particle uptake, it was shown that 3 × 3 × 0.5 μm^3^ cuboids, administered at a rate of 3 microchips per cell, displayed the highest cellular association (ca. 25%) and uptake (ca. 20%); therefore, this particle shape may have benefits for targeted delivery to macrophages compared to the other shapes used in this study. On the other hand, similarly sized cylinders and bar-shaped microchips displayed lower uptake of ca. 8% and ca. 6% and so may be beneficial for the avoidance of macrophage uptake. On average, 1–1.5 microchips were internalized, and ca. 1 microchip was surface-bound per cell, with cuboids showing the higher values. Overall, cuboids are the microchips at the front line for both cell surface bound and internalization, whereas thicker bars (0.5 μm) show the lower performance. As in the case of particle uptake, polysilicon microchips of different shapes did not elicit major changes in cellular metabolism, LDH release, or the release of lysosomal glucuronidase. In response to microchips, macrophages increased their metabolic activity and released low levels of intracellular enzymes, which could indicate a reduced stage of toxicity. Increasing the particle dose did appear to increase the metabolic activity of the cells, although it had no effect on enzyme release. Cuboids combine the attractiveness of exhibiting good uptake, at low doses, and lack of toxicity, positioning them as good candidates for drug delivery applications.

## Data Availability

Data will be made available upon request.

## Supporting Information Available

Additional experimental methods for cell culture; scattering intensity histograms (Figures S1 and S4); imaging flow cytometry images (Figures S2 and S5); plots correlating particle surface and MSI with association (Figures S3 and S6); plots correlating particle shape with internalized and surface-bound microchips (Figure S7); relationship between particle shape and LDH (Figure S8); and lysosomal glucuronidase (Figure S9) release (PDF)
