# Supplementary material for: Cuboids Prevail When Unraveling the Influence of Microchip Geometry on Macrophage Interactions and Metabolic Responses
Source: ACS Biomater Sci Eng. 2024 Aug 21;10(9):5689–700. doi: 10.1021/acsbiomaterials.4c00849 (PMC11388147; doi:10.1021/acsbiomaterials.4c00849)
Supplement: Supplementary file 1 — ab4c00849_si_001.pdf [file ab4c00849_si_001.pdf]

SUPPORTING INFORMATION

Cuboids Prevail when Unravelling the Influence of Microchip Geometry on Macrophage Interactions and Metabolic Responses

Gordon Bruce,[1] Saman Bagherpour,[2,3] Marta Duch,[4] José Antonio Plaza,[4] Snow Stolnik,[5] Lluïsa Pérez-García[1,2,3]*

[1] *Division of Advanced Materials and Healthcare Technologies, School of Pharmacy, University of Nottingham, Nottingham NG7 2RD, UK*

[2] *Departament de Farmacologia, Toxicologia i Química Terapèutica, Facultat de Farmàcia i Ciències de l'Alimentació, Universitat de Barcelona (UB), Av. Joan XXIII 27-31, 08028 Barcelona, Spain*

[3] *Institut de Nanociència i Nanotecnologia (IN2UB), Universitat de Barcelona (UB), 08028 Barcelona, Spain*

[4] *Instituto de Microelectrónica de Barcelona IMB-CNM (CSIC), Campus UAB, Cerdanyola del Vallès, Barcelona 08193, Spain*

[5] *Division of Regenerative Medicine and Cellular Therapies, School of Pharmacy, University of Nottingham, Nottingham NG7 2RD, UK*

1 Routine cell culture methods

1.1 Culture of RAW 264.7 cells

RAW 264.7 macrophages were cultured in T-75 cell culture flasks. 15 mL high glucose DMEM supplemented with 10% FBS, 1% L-glutamine, and 1% penicillin/streptomycin was used as the culture media. Cells were incubated at 37 °C with 5% CO_2 in 95% humidity. Cell culture media was removed by aspiration and replaced with 15 mL pre-warmed fresh media every 48 hours.

Cells were harvested by gentle scraping once they reached 60-80% confluency. Successful detachment of cells by scraping was confirmed by examination under a microscope. Detached cells in media were transferred by pipette into a 15 mL falcon tube. Falcon tubes containing cells were centrifuged at 1200 RPM (300G) for 5 minutes to cause the cells to form a pellet. The supernatant was removed by aspiration and replaced with 6 mL fresh (pre-warmed at 37°C) media. The cells were suspended in the fresh media by pipetting up and down to disperse the pellet. At this point cells would either be counted for seeding in well plates for experiments or if undergoing routine passaging would be split into new T-75 culture flasks containing 15 mL fresh pre-warmed media. Typical passaging ratios from 6 mL cell suspension were 1:3 and 1:6. Cells were passaged between P13 and P30 after which cells were disposed of and fresh P13 cells were revived from storage.

1.2 Cell counting, freezing, and revival

A haemocytometer was used to count the number of cells for each experiment. 10 μL detached cell suspension in fresh pre-warmed media was transferred to a 200 μL microcentrifuge tube. To this, trypan blue solution (0.4%, 10 μL) was added and mixed by pipetting the solution up and down. 10 μL of this mixture was transferred onto the

haemocytometer and cells counted under the microscope. Cells were counted from the four corners of the haemocytometer and the sum divided by four to give an average. This result was then multiplied by 2 to account for the dilution by adding the trypan blue solution and then multiplied by 10,000 to give the number of cells per 1 mL.

After detachment of cells and centrifugation, cells were suspended in 1 mL cell culture media containing 10% DMSO. This cell suspension was then transferred by pipette to a 1 mL cryovial. Cryovials were labelled with the date, cell type, and passage number and then placed in a Mr Frosty™ (Thermoscientific) freezing container containing isopropanol. The Mr Frosty™ container was placed in a -80 °C freezer overnight after which time cryovials were transferred into liquid nitrogen containers for long-term storage. Each cryovial contained a whole flask of cells.

After removal of a cryovial containing cells from liquid nitrogen storage, the cryovial was placed in a water bath at 37 °C until partially defrosted. As soon as the cell suspension had partially defrosted it was transferred to a 15 mL falcon tube and 10 mL pre-warmed media added. The suspension was centrifuged at 1200 RPM (300 G) for 5 minutes and the supernatant removed. The pellet was suspended in 15 mL fresh pre-warmed media and this cell suspension transferred to a fresh T-75 cell culture flask and stored in an incubator at 37 °C, 95% humidity, 5% CO_2.

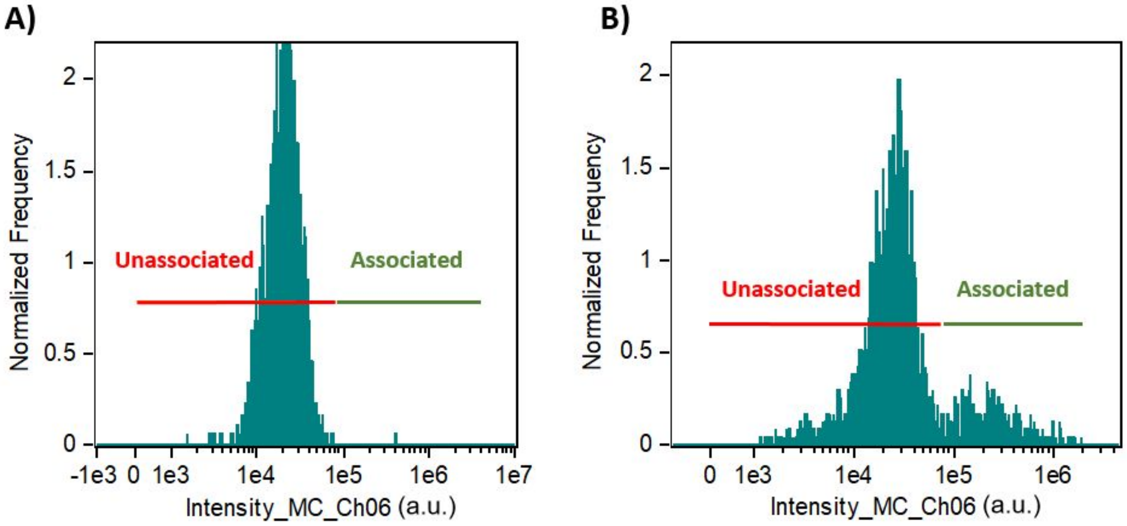

Figure S1. Distinction between polysilicon particle-associated and unassociated cells using scattering intensity. A) Scattering of untreated RAW 264.7 macrophages B) Scattering of RAW 264.7 cells treated with polysilicon Cuboids (3x3x0.5 μm)

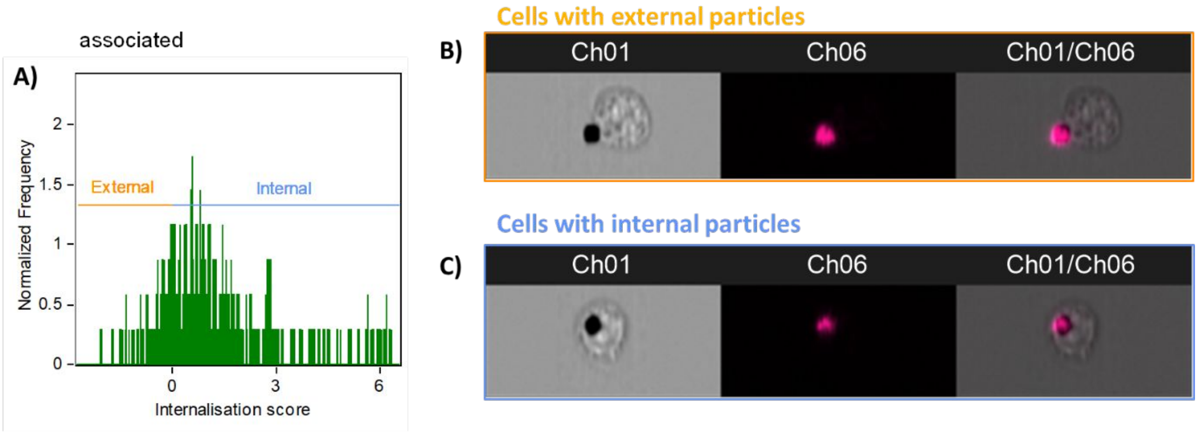

Figure S2. Distinction between cells with internal microchips and cells with external microchips A) Histogram showing internalisation score. Brightfield, scattering and overlay images of B) Cell with external particle C) Cells with internal particle

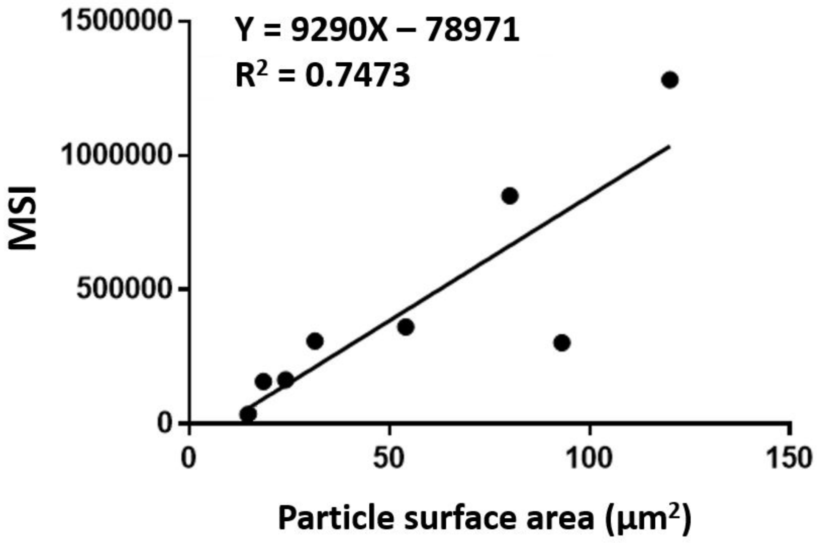

Figure S3. Correlation between MSI and particle surface area (R^2 = 0.75)

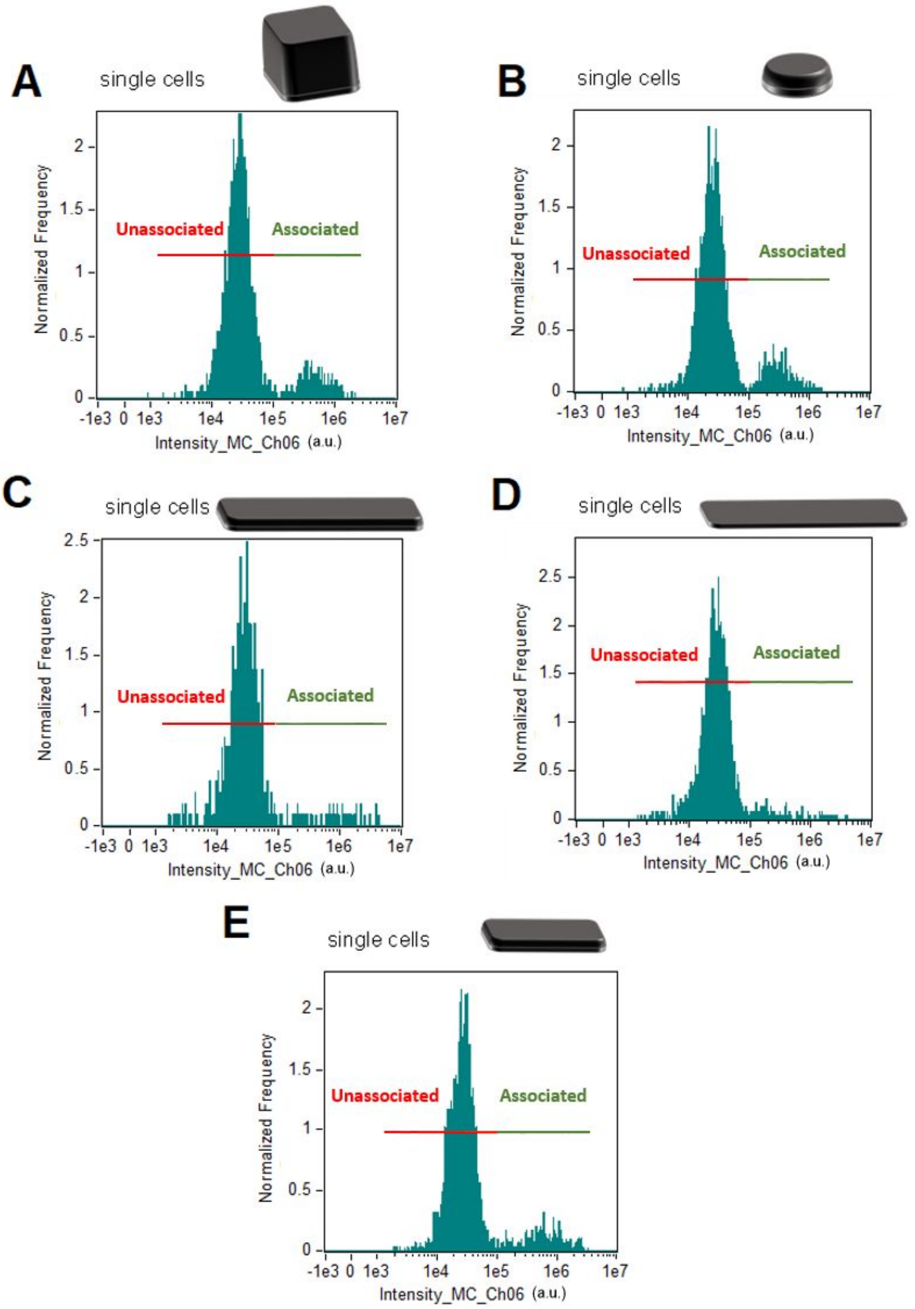

Figure S4. Side scattering intensity histograms of RAW 264.7 cells incubated with polysilicon microchips. A) Cubes (3x3x3 μm^3), B) Cylinders (d=3 μm h= 0.5 μm) C) Bars (3x15x0.5 μm^3), D) Bars (3x15x0.05 μm^3), E) Bars (3x10x0.5 μm^3).

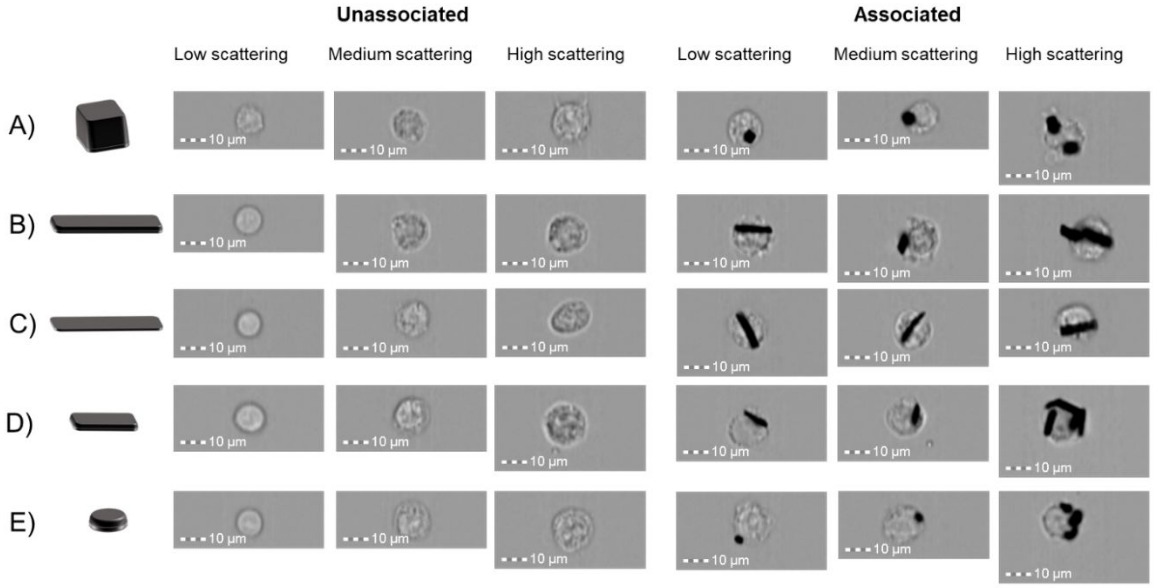

Figure S5. Cell images from imaging flow cytometry across the range of scattering intensities for cells non-associated and associated with polysilicon microchips. A) Cubes (3x3x3 μm^3), B) Bars (3x15x0.5 μm^3), C) Bars (3x15x0.05 μm^3), D) Bars (3x10x0.5 μm^3), E) Cylinders (d=3 µm h= 0.5 µm).

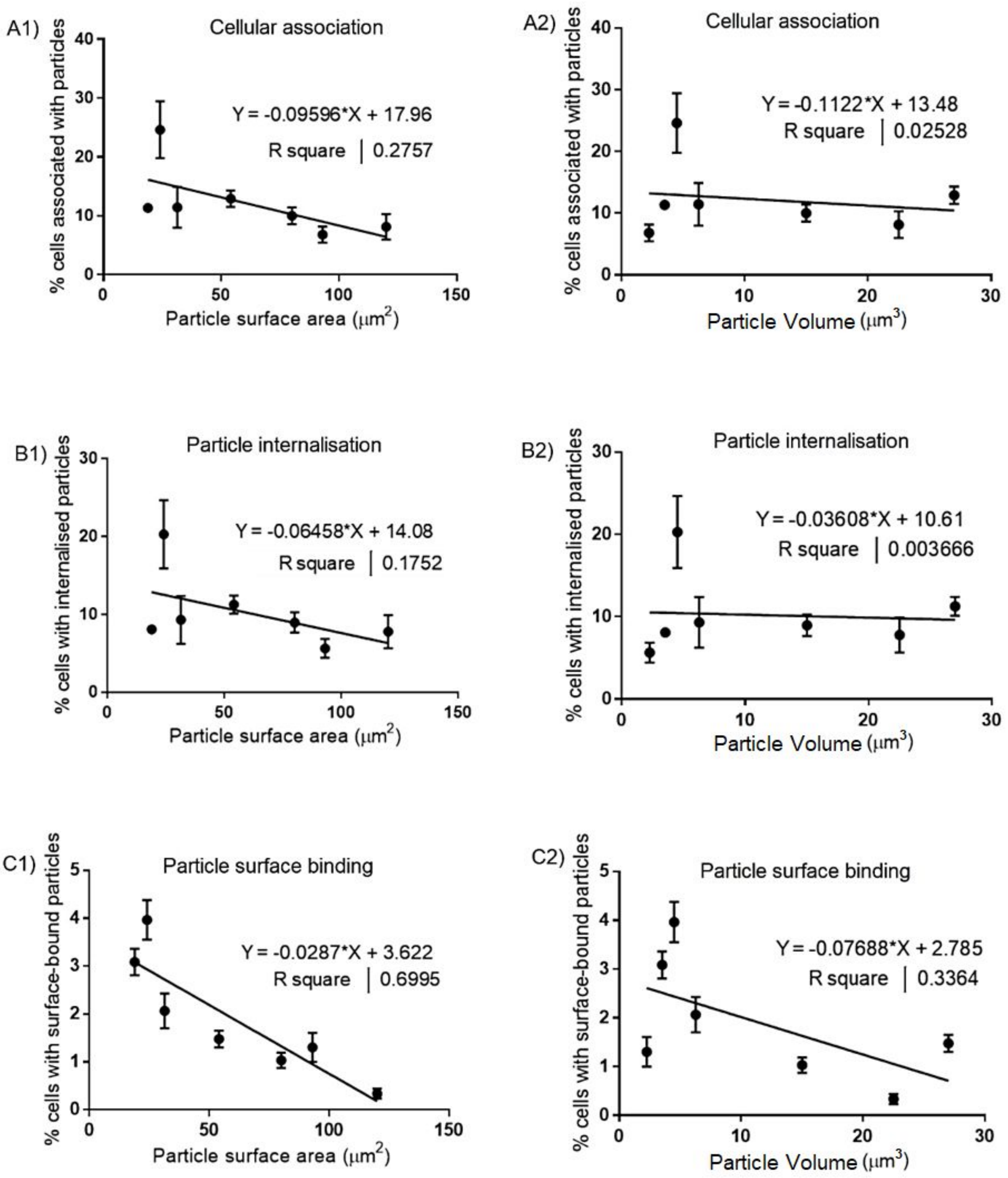

Figure S6. Particle factors affecting cellular association, internalisation, and surface-binding. Effect of particle surface area on A1) cellular association, B1) particle internalisation, and C1) particle surface binding. Effect of particle ovolume on A2) cellular association, B2) particle internalisation, and C2) particle surface binding

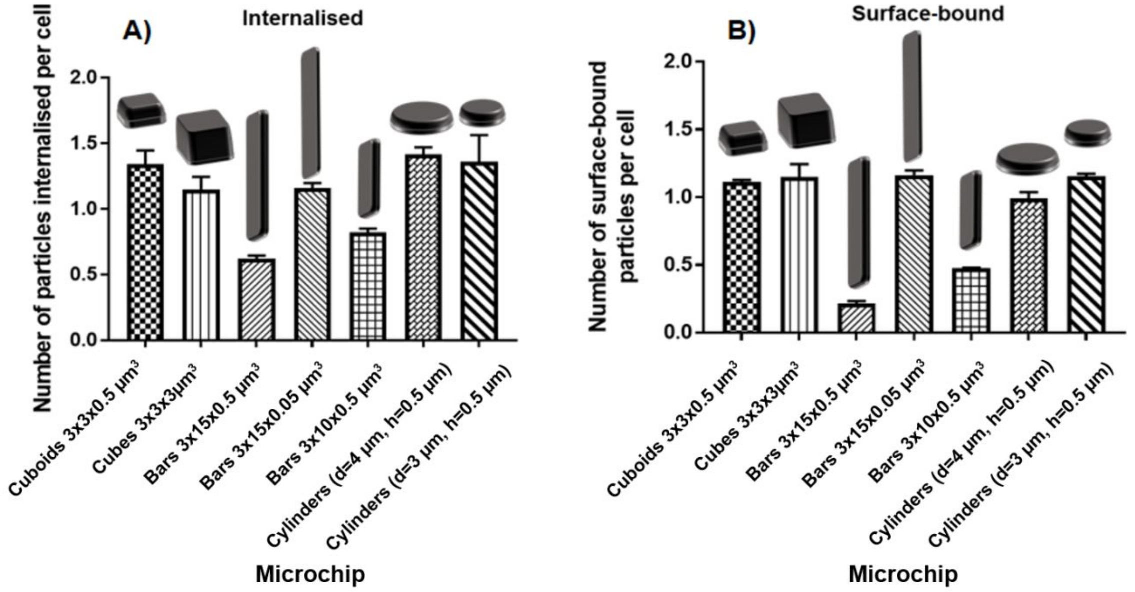

Figure S7. Effect of particle shape on number of A) internalised and B) surface-bound microchips per cell. Statistical differences analysed by one-way ANOVA with Tukey's multiple comparisons test (* $p<0.05$, ** $p<0.01$, *** $p<0.001$, **** $p<0.0001$). n=1, N=3 ± SEM

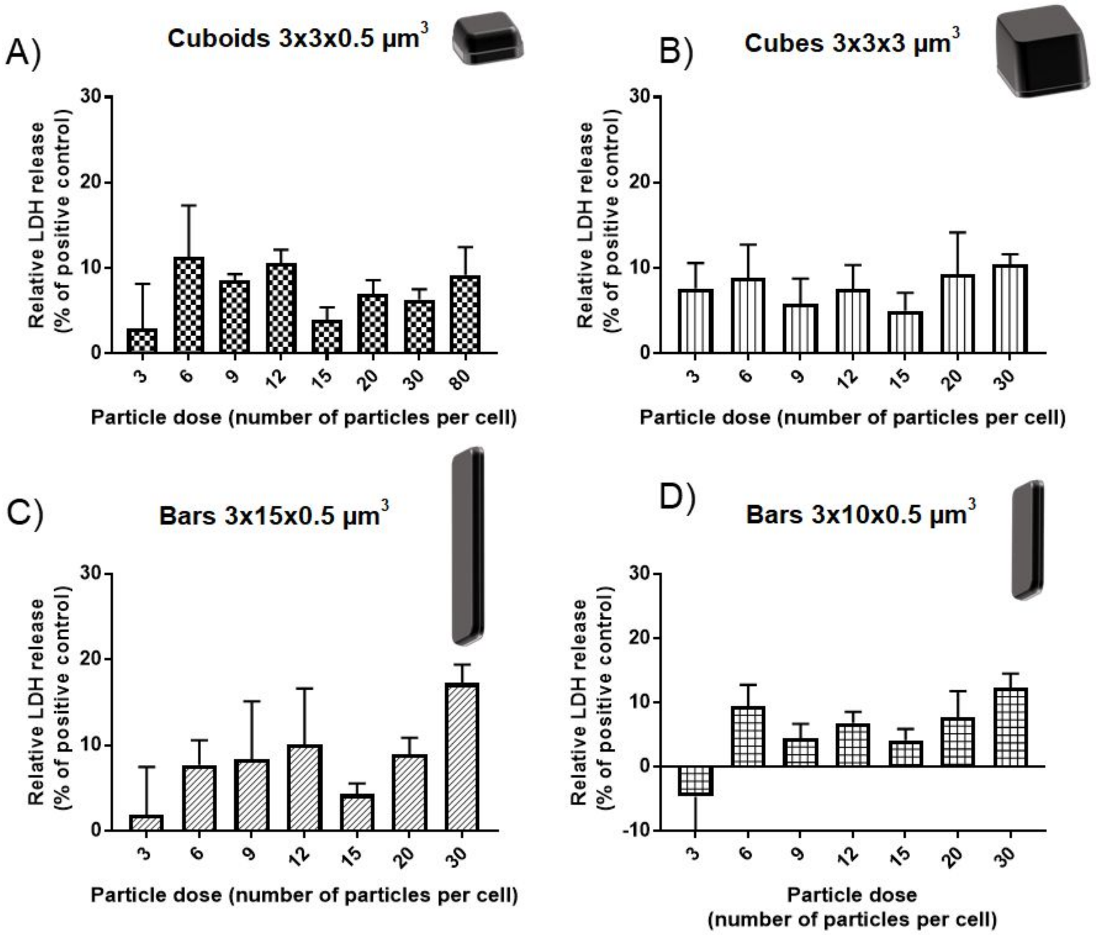

Figure S8. Effect of increasing polysilicon microchips dose on RAW 264.7 LDH release. A) Cuboids (3x3x0.5 μm^3), B) Cubes (3x3x3 μm^3), C) Bars (3x15x0.5 μm^3), and D) Bars (3x10x0.5 μm^3). Relative LDH release was calculated with respect to cells treated with 1% HEPES in HBSS (negative control), and cells treated with 0.1% Triton X-100 (positive control). Data is expressed as a mean ± SD (n=3).

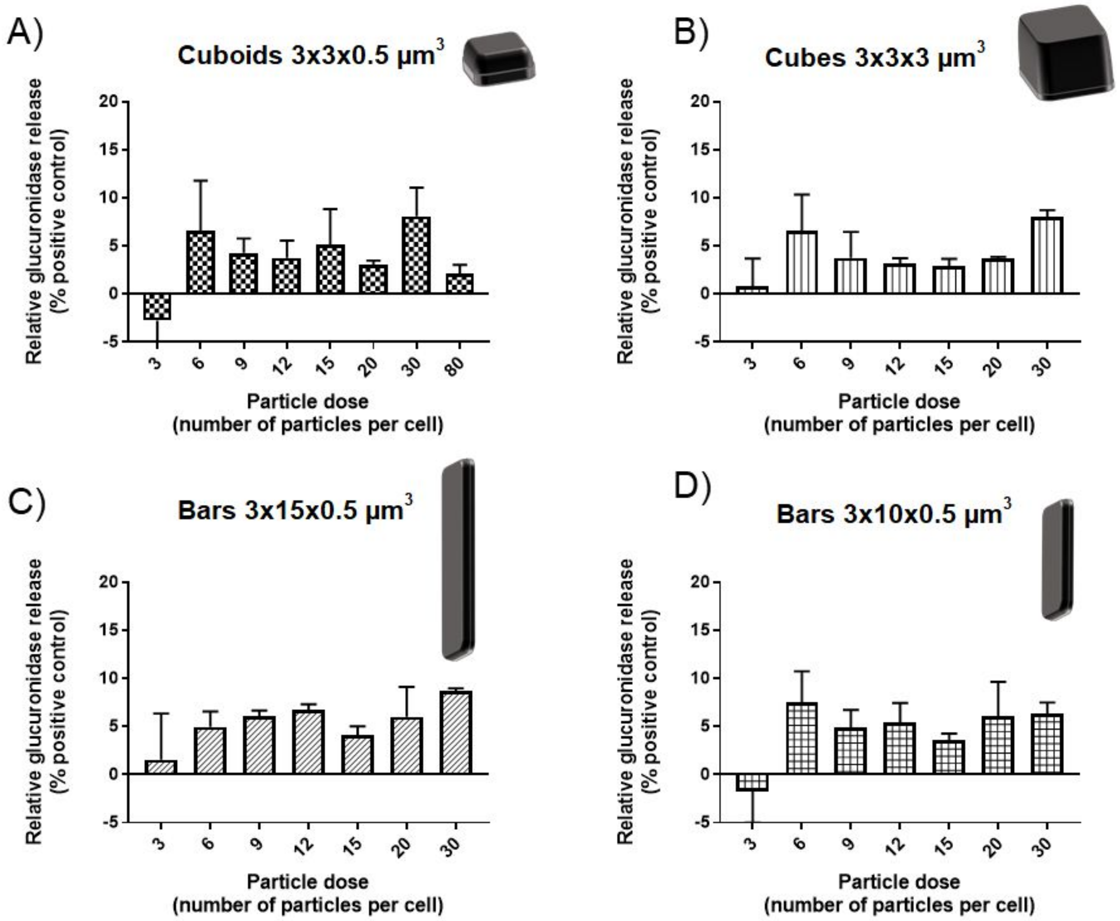

Figure S9. Effect of increasing polysilicon particle dose on RAW 264.7 lysosomal glucuronidase release. A) Cuboids (3x3x0.5 μm^3), B) Cubes (3x3x3 μm^3), C) Bars (3x15x0.5 μm^3), and D) Bars (3x10x0.5 μm^3). Relative glucuronidase release was calculated with respect to cells treated with 1% HEPES in HBSS (negative control), and cells treated with 0.1% Triton X-100 (positive control). Data is expressed as a mean ± SD (n=3).
